# Physiological and Therapeutical Effects of Tannic Acid

**Published:** 1850-11

**Authors:** 


### ECLECTIC DEPARTMENT,

### AND SPIRIT OF THE MEDICAL PERIODICAL PRESS.

*Physiological and Therapeutical Effects of Tannic Acid.* By Dr. Alison.

As an astringent, I have found tannic acid exceedingly efficacious, certainly as much so as any other agent, vegetable or mineral, that I have ever employed. It has equalled the salts of lead, copper, and zinc, without producing any of those poisonous effects which are liable to follow the free use of the salts of the first two metals.

*Internal Use.*—In the *chronic bronchial catarrh* of weakly and elderly persons, unconnected with disease of the heart or great blood-vessels, and attended with copious and debilitating expectoration, the administration of tannic acid by the mouth, in doses of one, two, and three grains, two or three times daily, has greatly and gradually abated the secretion, relieved the frequent cough, and improved the strength of the patient. In the second stage of *pulmonary consumption*—viz., that of softening, when bronchial catarrh has been present to a large extent, weakening the patient, causing frequent cough, and disturbing sleep, the same results have followed, and have greatly contributed to the comfort and welfare of the sufferer. But in pulmonary disease, the greatest amount of benefit has obviously been derived when large cavities have been present in the lungs, the walls of which have thrown out large quantities of purulent matter, occasionally mixed with blood. In such cases the discharge has been effectually controlled, and the rate of tear and wear of the system obviously restrained, without the induction of oppression or other evils.

In *chronic diarrhœa*, which had resisted the ordinary treatment by chalk, opium, and regulated diet, and was not dependent on obstructive disease of the heart or liver, tannic acid, in a solid form, has proved of surprising efficacy. In cases of severe disease, depending on irritable mucous membrane, I have not known of one failure; and of those examples connected with chronic inflammation and disorganization of the mucous membrane, only two proved beyond the influence of this remedy. These two cases occurred during the last autumn, while cholera was prevalent, and the disease of the mucous membrane was extensive. The complaint, in one of the examples, was of long standing, and the patient had been addicted to habits of intemperance. But it was not tannic acid only that failed; the salts of copper, iron, lead, and zinc, in large doses, proved to be of no more avail. In this form of disease tannic acid was administered in the form of pill, in combination with opium.

In *leucorrhœa*, unconnected with inflammatory action, I have found tannic acid efficacious in restraining the discharge, and in increasing the strength of the patient. The aqueous solution, combined with a small proportion of dilute nitric acid, was the form usually employed in these examples of disease. In *menorrhagia*, not dependent on a plethoric state of the system, or on local congestion, it was also serviceable, administered in the same form.

The excessive *sweating in phthisis*, and in other diseases running on to a fatal termination, has been usefully restrained by the use of tannic acid, combined with dilute nitric acid; and the habitual cold damp upon the skin of soft, weakly constitutions, has been corrected by the same means. I have had no opportunity of testing the virtues of this remedy in the *hemorrhagic diathesis;* but I am strongly disposed to believe they would be found very considerable, conjoined with other suitable means. I believe it would prove serviceable in *albuminuria*, dependent on chronic disorganization of the kidney, and not associated with obstructive disease. When the egress of albumen results, as I believe it often does in no small degree, from reduced tone and elasticity in the organ, and is not (as in a great majority of cases) a wholesome outlet necessary for the relief of the circulation, tannic acid offers the promise of benefit. Such a case, however, I have not lately met with, and consequently have not had an opportunity of testing the treatment.

*Local application.*—In the form of aqueous solution, used as a gargle, tannic acid has been most useful in correcting relaxation of the throat. Sponginess and hemorrhage of the gums have been greatly controlled by a lotion of tannic acid, and by the application of the dry powder. By this means loose teeth may be retained for a time, and the impediment to articulation thereby prevented, which would result from their removal.

In *prolapsus ani* I have prescribed tannic acid, dissolved in water, as an injection. This remedy is particularly indicated when the disease is associated with great relaxation of the solids. Applied to *hemorrhoidal tumors*, free from inflammation, in the form of a fine powder, mixed with lard, it would doubtless prove more efficacious than galls, the usual remedy. It is assuredly due to the tannic acid which it contains, that uva ursi proves serviceable in *catarrhus vesicæ.*

In *gonorrhœa*, chronic or about to become such, tannic acid, applied externally as a lotion, has proved serviceable. In the latter mode it has induced no smarting, although the parts have been tender, and though it has been applied with little intermission for several days. It is as a *local* astringent that tannic acid produces the most obvious effects, as Dr. Garrod has remarked.

Of tannic acid as an astringent, I have merely further to say, that it is of special excellence as an external application to the skin, when such a remedy is required. I have found it of extraordinary efficacy when reduced to a fine powder, mixed with lard, and applied to the skin. The parts soon acquire a healthy aspect; very little of the smarting or pungency is experienced, which so generally results from the use of the salts of alumina, lead, zinc, or copper. I have found it far superior to gallic acid. By way of testing their comparative powers, I lately employed an ointment of gallic acid to one spot of psoriasis, and one of tannic acid to another. The strength of both was the same. The spots were of old date, and had resisted much treatment. In the course of two days, the spot to which tannic acid had been applied was all but healthy; that for which gallic acid had been similarly employed was more enflamed than before. The gallic acid had caused smarting, and brought away the protecting scales. This treatment was adopted merely to test the comparative powers of the two acids, and not as curative practice. Astringents, if they be applied in psoriasis, must be used only as subsidiary to other treatment.

As a peptic, tannic acid is very efficacious. This I soon found, while employing it as a pure astringent. Symptoms of dyspepsia disappeared under its use ; the appetite increased, flatus and sense of distention were abated at the same time ; and in several instances the bowels, far from becoming constipated, acquiring a more healthy tone, actually became more free. A lady affected with phthisis, who has been under my care for three years, during which time she has taken tannic acid alternately with cod-liver oil, complained very lately of loss of appetite while taking the oil. The morning dose of the oil was replaced by tannic acid, combined with dilute nitric acid, and the result was a very striking restoration of the appetite. With such obvious improvement in the condition and action of the stomach, it is reasonable to believe that one of the results is the formation of a more perfect chyle. The action as a peptic is in accordance with the statement of one of the best writers on materia medica. Dr. Pereira says:—"Administered in moderate doses, they (astringents) promote the appetite, assist digestion," &c.

As a histogenetic, in promoting the *genesis*, and in *improving the quality of the blood*, tannic acid, it may be inferred from what has been stated above, would probably prove effective; but that it is really so, I have the evidence of improved complexion, greater fullness of the blood-vessels, increase of strength, buoyancy of spirits, and improved secretions, in numerous examples of anæmic and other diseases, in which this agent has been long employed.

The *formation of structures in the young*, I have reason to believe, is subserved, to a valuable extent, by the long-continued administration of tannic acid, in moderate doses. It is nearly six years since I began to prescribe this remedy in cases of curvature of the bones in children, with short shafts and enlarged epiphyses. The number of cases placed under this treatment while I was physician to the Northern Dispensary, was considerable, and not a few occurring in private practice were similarly treated. The general health was improved in all. The secretions, in many cases exceedingly offensive, were greatly corrected. In the course of a year or two, an obvious improvement in the shape and form of the bones was manifest. The curve was reduced, and the heads of the bones had lost no small amount of their disproportionate prominence. I have lately seen two or three children, presenting no appearance of having suffered from this affection of the bones, who some years ago really were deformed, and who were put under the influence of tannic acid, and also, it is true, of suitable regimen. In most of these examples of disease, when they came under my care, the urine contained an undue proportion of lime. This continued to be the case, at least for some time, even under the use of tannic acid, though perhaps not to the same extent. If tannic acid really possesses the power of correcting the tendency to rickets, or of staying the progress of this affection, it cannot be through any astringent action on the kidney arresting the exit of an undue quantity of lime, which is only a sign or consequence of the disease, and not its cause. It must act by invigorating the general health, and by imparting a more healthy character to the formative processes, by virtue of which lime and other mineral ingredients in the blood are more forcibly attracted to, and fixed in, the osseous structure. Further evidence of the power of tannic acid to improve the formation of tissues has been afforded by the increase in the volume and firmness of the soft parts of children placed under its operation, which I have frequently observed.

As a nervine of a lasting character, I have found tannic acid useful in several cases of nervous debility, langour, and excitability. These distressing conditions have been relieved; and the benefit, in one or two examples, has been permanent. Under the use of moderate doses of this medicine, I have known even the symptoms of weakly organization,—or, as I have thought, of impending softening of the brain, such as flightiness of speech and manner, impatience of attention or of application, hasty judgments, weakness and unstable gait,—to lose not a little of their prominence. It has always, however, been my object to guard against depending on this or any other such remedy where there has been good reason to suspect the presence of inflammatory action, even in a subdued form. When thus used as a nervine, tannic acid should generally be combined with camphor, hops, or hyoscyamus. The shower-bath has been employed, and the secretions have been attended to, at the same time. Thus exhibited, I believe that tannic acid, by improving the natural galvanic battery, if our brain and nerves may be so figuratively designated, will really, in many cases of feeble volition and muscular action, produce not a little of that benefit which has been sanguinely looked for from galvanism and electricity, and which, when obtained, has been so fleeting—at least in my experience.—*London Jour. Med. Ranking's Abstract.*

